# Auto-transplantation of whole rat ovary in different transplantation sites

**Published:** 2017-12-15

**Authors:** Somayeh Tavana, Mojtaba Rezazadeh Valojerdi, Hussein Eimani, Naeimeh Sadat Abtahi, Rouhollah Fathi

**Affiliations:** 1 *Department of Embryology, Reproductive Biomedicine Research Center, Royan Institute for Reproductive Biomedicine, Academic Center for Education, Culture and Research (ACECR), Tehran, Iran; *; 2 *Department of Anatomy, Faculty of Medical Sciences, Tarbiat Modares University, Tehran, Iran; *; 3 *Department of Anatomy, Faculty of Medicine, Baqiyatallah (AJ) University of Medical Sciences, Tehran, Iran.*

**Keywords:** Auto-transplantation, Rat, Transplantation site, Whole ovary

## Abstract

This study was carried out to assess the different ovarian transplantation sites after short-time autografting. Female rats were randomized into five groups, with six rats in each group, including control (intact), cervical subcutaneous transplanted (CST), back subcutaneous transplanted (BST), subfascial transplanted (SFT) and intramuscular transplanted (IMT) groups. In all experimental groups, the right ovary was removed and transplanted into different sites. After three weeks, ovaries were removed for morphology assessment, follicular counting and the rates of corpus luteum (CL) and cyst formation. Transplanted ovaries in BST and SFT groups were full of cysts and did not have sufficient numbers of intact follicles and were excluded from experiments. In IMT and CST groups, re-anastomosis, follicular development and good homogeneity of the stromal tissue were seen. However, the difference in intact antral follicles between CST (7.92 ± 0.02%) and CST-Op (opposite ovary of CST group) (30.99 ± 0.03%) was significant as well as the difference between CST (7.92 ± 0.02%) and control (10.08 ± 0.01%) groups. In addition, the number of intact primordial follicles in the CST-Op (16.58 ± 0.02%) group was significantly less than that of the control (40.40 ± 0.03%) group. Interestingly, the number of CL was significantly increased in the CST-Op (11.71 ± 0.01%) and IMT-Op (9.16 ± 0.02%) groups compared to the control and experimental groups. Although both intramuscular and subcutaneous sites effectively preserved ovarian follicles after three weeks, cervical subcutaneous site was better suited for auto-transplantation in rat.

## Introduction

Ovarian transplantation is the only possible method for fertility preservation in prepubertal patients who do not possess an activated hypothalamus-hypophysis-ovarian axis.^1^^,^^2^ Although the ovarian transplantation procedure dates back to the 18^th^ century, its application for cancer patients was realized just recently.^3^

Many researchers have shown that grafted ovaries could restore reproductive and endocrine functions. Ovary transplantation techniques can be used to help patients unable to conceive naturally and to gain fertility potential.^4^ An important fact to be considered during transplantation is the reduction of ischemia-re-perfusion time.^5^ Since the rate of ischemia depends on the duration of re-anastomosis, the vascular condition of the transplantation site plays an important role in reduction or induction of this time.^6^

The choice of the transplantation site is an essential factor which plays an important role in the future grafts viability and the subsequent oocyte competence. Ovarian tissue can be transplanted back to the original site (orthotopic) or to alternative sites (heterotopic).^7^ For each site, clinical concerns such as the chance of natural conception, simplicity of the procedure, and easy access for oocyte retrieval must be taken into consideration.^8^

In the heterotopic transplantation, the tissue is grafted into other sites of the body such as under skin of arm and abdomen, peritoneal cavity, abdominal rectus muscle, gluteus, deltoid muscles, omentum, under kidney capsule, mesovarium, stomach wall, and axillary cavity.^9^^-^^14^ Despite these studies, ovarian tissue transplantation is yet in its experimental stage, and it still remains unknown which site is most optimal and practical.^15^

The aim of this study was to compare the different transplantation sites to find the optimal position for whole ovarian auto-transplantation in a rat model.

## Materials and Methods

**Chemicals and animals. **All chemicals were obtained from Sigma except otherwise indicated. The study’s ethical approval was obtained from the Ethical Committee of the Royan institute (EC/90/1002, 2011). Five-week-old female rats (Wistar) were sourced from Pastor Institute (Tehran, Iran). The animals underwent 12 hr dark and 12 hr light time period in the laboratory animal unit. The rats were randomized into the five groups with six rats in each group, including control 1 (intact five weeks old rats) group, cervical subcutaneous transplanted (CST) group, back subcutaneous trans-planted (BST) group, subfascial transplanted (SFT) group and intramuscular transplanted (IMT) group. After transplantation, the opposite ovaries (CST-Op and IMT-Op groups) of each transplanted group was immediately fixed in fixative solution for subsequent analyses. Also, ovaries were removed from control 1 group after three weeks, considered as control 2 (eight weeks old rats) supplementary group.

**Auto-transplantation. **Before surgery, each rat was anesthetized with the intra-peritoneal injection of 50 mg kg^-1 ^ketamine (Alfasan, The Netherlands) and 5 mg kg^-1 ^xylazine (Alfasan) and then the rat was prone on the work bench.^16^ In each rat, the skin over the transplantation site and right ovary region was completely shaved and opened with a surgical blade. The right ovary was removed and separated from oviduct and fat pad including vessels and nerves with a cutter. The removed ovary was immediately placed in the site. In animals of CST group, the skin on the back of the neck was opened and the ovary was transplanted into the subcutaneous tissue. A little sore was made in the cervical erector spine muscles for bleeding. In the BST group, the skin of the right flank was opened and the procedure was similar to that described earlier. For SFT group, after opening the skin of gluteal region and the fascia covering the gluteus muscle, the removed ovary was laid under the deep fascia (fascia covering the gluteus muscle). The fascia was then closed with a little suture. The ovary in the IMT group was transplanted to the opened gluteus muscle on the right side. The muscle and skin were then closed with a little suture. All animals were placed in the separate cages for three weeks. 

**Transplanted tissue removal. **Three weeks after transplantation, eight-week-old transplanted rats were anesthetized again. After shaving the skin and opening the site, the transplanted tissue was separated from adjacent recipient tissue with a safe margin and was fixated in Bouin’s solution (manually prepared) for the following analyses.

**Histology. **All fixated ovaries were dehydrated and embedded in paraffin wax, serially sectioned at 6 μm and stained with hematoxylin and eosin. Five slides (each one containing eight sections) from each sample were analyzed using a light microscope and the numbers of morphologically normal and atretic follicles were counted according to Tavana *et al*., in which the follicles were classified as: primordial (an oocyte surrounded by a single layer of flattened pregranulosa cells), primary (an oocyte surrounded by a layer of cuboidal granulosa cells), preantral (an oocyte surrounded by two or more layers of granulosa cells with no antrum), and antral (when the antral cavity was observed) follicles.^17^

**Statistical analysis. **The SPSS software (version 16; SPSS Inc., Chicago, USA) was used for data analyses. All data were normalized by Kolmogorov-Smirnov as a nonparametric test before analysis. The number of morphologically intact and atretic follicles and the number of corpora lutea and cysts were compared using ANOVA test among the groups followed by post hoc Tukey tests. Data were presented as the mean ± SEM, and differences with *p *< 0.05 were considered statistically significant.

## Results

**Fig. 1:**
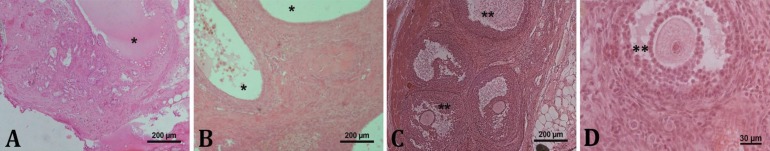
Ovarian tissue sections stained with hematoxylin and eosin (H & E) three weeks after ovary transplantation in four different sites. A) Back subcutaneous transplant (BST), B) Subfascial transplant (SFT), C) Intramuscular transplant (IMT), D) Cervical subcutaneous transplant (CST). * ^,^ ** illustrate cysts and large antral follicles, respectively.

**Morphology and follicular count. **After tissue sectioning and observing the morphology of transplanted tissues, it was seen that there were no healthy follicles in the groups of BST and SFT. They were full of cysts and fibrotic tissues and low numbers of follicles. Thus, these two groups were eliminated from the study (Fig. 1).

The numbers of primordial follicles in all experimental groups were similar to those of controls 1 and 2 and there was no significant difference except between CST-Op group and both control groups. There was also no statistical difference in the number of atretic primordial follicles among all controls and transplanted ovaries. In the primary follicle, the largest quantity of intact follicles was for CST group and the smallest quantity of intact follicles was for the CST-Op group. However, there was no statistical difference among all groups. The number of atretic primary follicles was the lowest in control group and this was significant compared to both CST and IMT groups.

The number of intact preantral follicles was high in both CST-Op and IMT-Op groups. The intact preantral follicles number was the lowest in IMT group which was differed significantly from control 1, control 2 and IMT-Op groups. Atretic preantral follicles were similar in number among all groups. Growing of follicles to the antral stage had the highest rate in CST-Op and IMT-Op groups. This growth rate was significantly different in CST-Op compared to control 1, control 2 and CST groups. The number of atretic antral follicles was statistically less in CST-Op than those in control 2, CST and IMT transplanted groups. Furthermore, the difference in the number of atretic antral follicles in CST group was significant compared to CST-Op group (Fig. 1 and Table 1).

**Table 1:** Number of morphologically intact and dead follicles in intact (control), transplanted and opposite ovaries. Data are presented as the mean ± SEM

**Groups**	**Primordial**		**Primary**		**Preantral**		**Antral**		**AFI**
	**Intact**	**Dead**	**Intact**	**Dead**	**Intact**	**Dead**	**Intact**	**Dead**	**Antral/Preantral**
**Control 1**	38.53 ± 0.03^c^	2.91 ± 0.00	26.20 ± 0.03	4.26 ± 0.00^A^	10.01 ± 0.01^a^	2.49 ± 0.00	12.86 ± 0.02^a^	2.70 ± 0.00^A^	1.30
**Control 2**	36.44 ± 0.04^c^	4.60 ± 0.02	27.05 ± 0.03	7.79 ± 0.03	11.33 ± 0.02^a^	2.33 ± 0.01	8.26 ± 0.04^a^	0.4 ± 0.02^a^	0.73^a^
**CST**	35.63 ± 0.04^c^	4.97 ± 0.00	27.60 ± 0.04	11.53 ± 0.03^a^	7.46 ± 0.04	3.16 ± 0.01	7.91 ± 0.02^a^	0.56 ± 0.00^aB^	1.06
**IMT**	34.99 ± 0.05	5.92 ± 0.02	24.66 ± 0.03	14.53 ± 0.02^a^	4.58 ± 0.01^A^	4.20 ± 0.02	9.56 ± 0.01	0.38 ± 0.00^a^	2.08^A^
**CST-Op**	16.57 ± 0.04^C^	2.16 ± 0.03	17.11 ± 0.19	5.94 ± 0.01	19.63 ± 0.31	4.68 ± 0.14	30.99 ± 0.67^A^	2.88 ± 0.03^b^	1.60^A^
**IMT-Op**	31.97 ± 0.55	1.42 ± 0.05	25.45 ± 0.32	7.12 ± 0.03	14.66 ± 0.33^a^	1.42 ± 0.15	16.08 ± 0.11	1.83 ± 0.02	1.10

Control 1: non-transplanted ovaries from five weeks old rat; Control 2: non-transplanted ovaries from eight weeks old rat; CST: subcutaneous cervical transplanted ovaries; IMT: intramuscular transplanted ovaries; CST-Op: Opposite ovaries of CST group; IMT-Op: Opposite ovaries of IMT group; AFI = Antral formation ‎index.

The differences in each column between capital and small letters are significant.

**Corpus luteum (CL) and cyst formation. **Although the number of CL was low in both CST and IMT transplanted groups, these were not significant different compared to control 1 and 2. The highest number of CL was observed in the opposite ovaries (CST-Op and IMT-Op groups). There was no CL formation in both BST and SFT groups.

The cyst formation rates were high in both BST and SFT. These rates were significant with all control and experimental groups. The cyst formation rates in CST and IMT transplanted groups were significantly differed from the control 1 and 2, CST-Op and IMT-Op groups (Table 2).

**Table 2:** Corpus luteum and cyst formation in intact (control), transplanted and opposite ovaries. Data are presented as mean ± SEM.

**Groups**	**Corpus luteum**	**Cyst**
**Control 1**	3.47 ± 0.44^ab^	0.00^ab^
**Control 2**	4.70 ± 0.30^ab^	0.00^ab^
**CST**	1.12 ± 0.70^a^	3.67 ± 0.55^A^
**IMT**	1.14 ± 0.50^b^	2.60 ± 1.07^B^
**CST-Op**	11.70 ± 2.72^A^	0.67 ± 0.33^a^
**IMT-Op**	9.16 ± 4.58^B^	0.00^b^
**BST**	0.00	12.00 ± 1.00
**SFT**	0.00	10.00 ± 1.00

Control 1: non-transplanted ovaries from 5 weeks old rat; Control 2: non-transplanted ovaries from 8 weeks old rat; CST: subcutaneous cervical transplanted, IMT: intramuscular transplanted; CST-Op: Opposite ovaries of CST, IMT-Op: Opposite ovaries of IMT; BST: back subcutaneous transplanted, SFT: subfascial transplanted.

The differences in each column between capital and small letters are significant.

## Discussion

In general, there are two main approaches for ovarian tissue auto-transplantation, orthotopic and heterotopic. Although normal pregnancy is one of the benefits of orthotopic transplantation, this procedure needs abdominal surgery and general anesthesia. Moreover, when the risk of the ovarian metastasis is high, orthotopic transplantation is not preferred because monitoring of the malignant cells in the tissue is very difficult. Thus, in order to reduce surgery risks, and to enable monitoring and frequent egg retrieval from graft, heterotopic transplantation is preferred over orthotopic methods. An added advantage of heterotopic transplantation is that the procedure can reestablish endocrine function. However, the body region that is best suited for ovarian tissue grafting is not yet known.^18^

In this study, we examined the growth of follicles and CL and cyst formation in four sites – intramuscular (IMT), subfascial (SFT), back subcutaneous and (BST) cervical subcutaneous (CST) sites - after three weeks of rat ovarian auto-transplantation. The results of histology evaluation showed the existence of follicles in different stages especially ovulatory follicles which approved active folliculogenesis in the graft. Because of the low numbers of follicles and large numbers of cysts and fibrotic areas in the grafted tissue, SFT and BST groups were excluded from the study. Therefore, the present study was carried out using the IMT and CST groups.

The results showed that the SFT and BST sites exhibited similar behavior in terms of follicular count and ovarian tissue morphology as the control group and in general there were no statistical differences in the number of follicles. The growth rates of primordial follicles were generally lower in CST, IMT, CST-Op, IMT-Op and control 2 than control 1, which indicates the consumption of follicular reserve, an expected phenomenon in all animal species. The reduction of early stage follicles and increased death of primordial follicles in control 2 more than control 1 confirmed this idea. 

Primordial follicles, due to their ability to grow in hypoxic condition and low metabolism, are resistant to ischemia. Consequently, primordial follicles are preserved better than the other types of follicles during transplantation. This idea suggests that inhibitory factors secreted from antral follicles could keep primordials in dormancy;^19^ however, it is not in consistence with our data from the present study. In control 1, control 2, CST and IMT groups, despite the low number of antral follicles primordials were of maximum count. However, in CST- Op group the primordials count was the lowest versus antral follicles that were found in large numbers among the other groups. In the present study the primordial, primary and antral follicles showed no significant decrease. However, there was a significant reduction in the number of preantral follicles in the intramuscularly grafted (IMT) group compared to controls 1 and 2. 

A high percentage of follicular loss after trans-plantation may be due to the lack of nutrients caused by delayed re-angiogenesis.^20^ In both CST and IMT groups, primordial follicles were preserved after three weeks of transplantation, same as the controls 1 and 2. Li *et al*. showed that follicular death occurs shortly after transplantation, leading to loss of follicles in the early stage of development.^20^ Since primordial follicles are more resistant to ischemic conditions, they could be constant in number. Callejo *et al*. reported that ischemia induces death in the follicular reserve during transplantation.^21^ In our study, atretic primary follicles were observed in the IMT and CST groups especially when compared to the control 1. The significant differences could be attributed to ischemia after transplantation. A remarkable fact is that the occurrence of primary follicle death was higher than the other follicular stages in control and OP groups, however, this follicle death occurrence was increased significantly in transplanted groups.

Although primary follicular death was increased in transplanted groups, follicular growth was compensated from preantral to antral stage (especially in IMT group) which indicated that antral follicle formation has been facilitated during transplantation. Antral follicle formation in opposite ovaries, especially CST-Op group, showed a conspicuous increase compared to the others groups, and this could be related to the activity of the hypothalamus-pituitary-ovarian axis.

The existence of CL in transplanted ovaries indicates ovulation and is a sign of reactivity of the ovary after transplantation.^22^ The highest number of CL were seen in the ovary of Op group, because of the lack of injury in pituitary–ovary axis.^23^ Furthermore, because of debilitation in pituitary–ovarian correlation, CLs were reduced clearly in transplanted ovaries compared to Op groups. In rodents usually luteolysis is induced by PGF2α that is produced from corpora lutea in the other ovary. In some animal species, the uterus is the main source for PGF2α but in rats, only the CL produces PGF2α.^24^ In the other words, because of transposition of the grafted ovary to a site far from the original position, the interaction between two ovaries is weakened. Thus, the luteolytic factors secreted by the grafted tissue cannot reach the non-transplanted ovary that remains in the original location.

Cyst formation in transplanted ovaries was found to be significant. The main reason for this phenomenon is the rate of re-anastomosis. In the intramuscular site (IMT group), the ovary lies inside full vascularized muscle fibers and several vascular networks. Therefore, the rate of re-anastomosis was more than that of ovaries transplanted to the cervical subcutaneous region (CST group), which was only served by the subcutaneous plexus. In this area, due to delayed re-anastomosis, fibrotic tissues were increased around the ovary especially in tunica albuginea. This dense connective tissue did not permit follicular rupture and the follicles that not releasing their oocytes, were changed to the follicular cyst, a type of functional ovarian cysts.

Callejo *et al*. found a high rate of cortical fibrosis after subcutaneous and intraperitoneal fresh ovarian trans-plantation in rats, which may be due to ischemic stress and delayed revascularization of the tissue and this supports the above conclusion as well.^21^ In the ovaries grafted in both SFT and BST sites, CL was not seen. Many cysts were also observed, which might be due to fluid accumulation in the graft. Cysts may cause repositioning of the transplanted tissue and does not allow effective attachment. The grafted ovaries in SFT and BST sites were excluded from the study not due to cyst formation or non-corpus luteum appearance but because of the presence of very low number of follicles. Presence of epithelial cysts, tissue fibrosis and reduction of initial follicles in heterotopic ovarian transplantation without vascular anastomosis has also been observed even after an extended period (six months and one year after transplantation) in a study by Callejo *et al*.^21^

The role of the location of transplantation on follicle survival and growth within the graft as well as on the quality of eggs retrieved is very critical especially during human ovarian re-implantation after cryopreservation and thawing. Because of inconsistent results, the site best suited for clinical application has not yet been found.^19^ Fast and easy re-angiogenesis occurs around the transplanted tissue and its surgical treatment causes folliculogenesis recycling and prevents ischemia injury. 

In recent years, subcutaneous and intramuscular sites have been introduced as alternative regions for ovarian transplantation. Although the kidney capsule has shown interesting results in ovarian transplantation outcomes, transplanting to this region involves difficult surgical and monitoring procedures.^20^ In spite of some limitations such as poor angiogenesis and vessel regeneration around the tissue,^20^ subcutaneous transplantation has been introduced and deployed as a common method in ovarian transplantation researches, because of simplicity in surgery and graft monitoring.^19^

Additionally, the graft under subcutaneous region is susceptible to temperature fluctuation, atmospheric pressure, and mechanical contacts.^20^ Since follicles grow and develop in sizes only up to 15 mm,^19^ temperature variations can cause damage to the cytoskeleton and mitotic spindle in subcutaneous grafts.^25^

It has recently been demonstrated that healing granulation tissue^6^ is a favorable alternative to ovarian grafts. Previous studies have shown a high rate of ovarian recycle after transplantation into the dorsal muscle.^26^

In a study, the dorsal muscle that mimics ovarian conditions was chosen as a graft site.^27^ This area also provides an environment rich in blood vessels and very little fibrotic tissue.^19^ Another advantage is that intramuscular site is more suitable for manipulation than the other sites.^28^

Considering the follicular reserve and ovarian integrity after short-term transplantation of frozen-thawed human ovarian tissue, four transplantation sites including intraperitoneal, ovarian bursa, subcutaneous and intramuscular regions, show similar properties.^29^

In conclusion, heterotopic autografting into both intramuscular and cervical subcutaneous sites in the rat model showed promising results. Although these sites effectively preserved ovarian follicles after three weeks, easier oocyte monitoring and retrieval under the cervical skin makes it more practical for auto-transplantation.
